# The Effect of Carnosine Supplementation on Depressive Symptoms and Health‐Related Quality of Life in Individuals With Prediabetes and Well‐Controlled Type 2 Diabetes: A Randomized Controlled Trial

**DOI:** 10.1002/fsn3.71514

**Published:** 2026-02-25

**Authors:** Robel Hussen Kabthymer, Jack Feehan, Aya Mousa, Giancarlo Aldini, Maximilian de Courten, James Cameron, Barbora de Courten

**Affiliations:** ^1^ Department of Medicine, School of Clinical Sciences Monash University Clayton Victoria Australia; ^2^ School of Health and Biomedical Sciences RMIT University Bundoora Victoria Australia; ^3^ Monash Centre for Health Research and Implementation (MCHRI), Faculty of Medicine, Nursing and Health Sciences Monash University Clayton Victoria Australia; ^4^ Department of Pharmaceutical Sciences University of Milan Milan Italy; ^5^ Institute for Health and Sport Victoria University Footscray Victoria Australia; ^6^ Monash Cardiovascular Research Centre, Monash Heart, Monash Health Clayton Victoria Australia; ^7^ Department of Diabetes & Vascular Medicine Monash Health Clayton Victoria Australia

**Keywords:** carnosine, depression, diabetes, histidine containing dipeptide, prediabetes, quality of life

## Abstract

Type 2 diabetes mellitus (T2DM) is commonly associated with mental health disorders such as depression and anxiety. Carnosine, an over‐the‐counter food supplement, may improve depressive symptoms through its anti‐inflammatory properties; however, its effects on depressive symptoms and quality of life in prediabetes or T2DM remain unexplored. This randomized controlled trial aimed to examine whether carnosine supplementation may improve depressive symptoms and quality of life among individuals with prediabetes and well‐controlled T2DM. A total of 38 participants (73.6% male) with a median (IQR) age of 54.8 years (46.2, 59.4) and mean ± SD body mass index (BMI) of 29.0 ± 4.2 kg/m^2^ were randomized to carnosine (*n* = 18) or placebo (*n* = 20) for 14 weeks. None of the patients were diagnosed with depression or anxiety or any other chronic disease other than prediabetes (*n* = 20, 52.6%) and T2DM (*n* = 18, 47.4%), the latter being well‐controlled with diet or metformin only. Depressive symptoms were measured using the patient health questionnaire (PHQ‐8) and health‐related quality of life was measured with five‐dimension EuroQoL three level (EQ‐5D‐3L) scale. Paired *t*‐tests were employed for within‐group comparisons. Analysis of covariance (ANCOVA) was used for between group comparisons adjusted for age, BMI, and baseline values. Carnosine supplementation resulted in improvement of depressive symptoms assessed by total PHQ‐8 score (mean difference = −2.0; 95% CI: −3.9, −0.2; *p* = 0.03), compared with placebo. However, the eight subcomponents of the PHQ‐8 scale did not show significant changes (*p* > 0.05). There were no significant changes both in between‐group and within‐group comparisons in health‐related quality of life scores (*p* > 0.05). We demonstrated for the first time that carnosine supplementation resulted in a modest improvement in depressive symptoms in individuals with prediabetes or T2DM. Further studies are needed to corroborate these findings in larger cohorts with more diverse baseline risk profiles.

**Trial Registration:** NCT02917928

## Graphical abstract

Carnosine supplementation resulted in a modest reduction of depressive symptoms but no change in health related quality of life in individuals with prediabetes or diabetes.

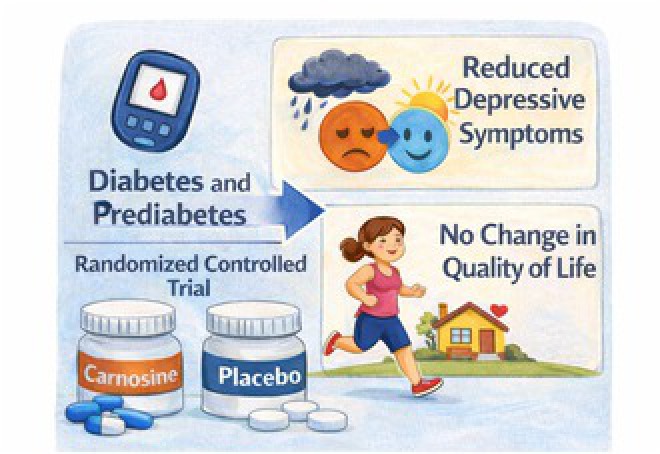

## 1 Introduction

Globally, approximately half a billion individuals live with diabetes, with type 2 diabetes mellitus (T2DM) accounting for 90% of the disease burden (IDF 2021). The global prevalence of prediabetes measured with impaired glucose tolerance (IGT) was 9.1% (464 million) in 2021 and is projected to increase to 10.0% (638 million) by 2045 (Rooney et al. 2023). The global prevalence and incidence of T2DM and prediabetes are high and expected to rise, mainly driven by obesity, sedentary lifestyles, and unhealthy diets (IDF 2019; Rooney et al. 2023). An estimated 1.3 million Australians, approximately one in twenty people, currently live with diabetes (Australian Institute of Health and Welfare 2022).

Globally, more than 970 million individuals are affected by mental health disorders, depression and anxiety being the most common (GBD 2019 Mental Disorders Collaborators 2022). Individuals with T2DM have a higher risk of mental health disorders (Arias et al. 2022; Deshpande et al. 2008; Ducat et al. 2014), with the prevalence of depression reported to be two‐fold higher in those with T2DM compared with the general population (Roy and Lloyd 2012). Similarly, the prevalence of depression is moderately increased in individuals with prediabetes compared with their normo‐glycaemic counterparts (Chen et al. 2016). This increased risk may be partly due to diabetes‐related distress, which affects a large proportion of this population and can contribute to depressive symptoms (Perrin et al. 2017). However, the precise mechanisms underlying these associations remain poorly understood. Recently, chronic low‐grade inflammation has been recognized as a potentially key pathophysiological factor linking metabolic and mental health disorders (Dowlati et al. 2010; Howren et al. 2009; Kohler et al. 2017; Osimo et al. 2019). Treatments which target the underlying inflammatory pathways may therefore improve mental health outcomes in populations with prediabetes or T2DM.

Carnosine is a histidine‐containing dipeptide that is naturally produced in humans, with known antioxidant and anti‐inflammatory properties (de Courten et al. 2016; Schon et al. 2023). Meta‐analysis of randomized clinical trials have reported that histidine‐containing dipeptide supplementation significantly reduces serum inflammatory biomarkers such as C‐reactive protein, tumor necrosis factor‐ alpha (TNF‐α), and malondialdehyde (MDA) (Saadati et al. 2023). In addition, carnosine supplementation also reduces the formation of advanced glycation and lipoxidation end‐products (AGE/ALE) and protein crosslinking (Aldini et al. 2021; Freund et al. 2018). The role of carnosine in glucose control is suggested to be via preventing the formation of AGE/ALE and destructive carbonyl species MDA and 4‐hydroxynonenal (HNE), which are suggested to be part of the pathogenic mechanism of T2DM (Manohar et al. 2013; Zarkovic 2003).

In our recent meta‐analysis of randomized clinical trials on the effects of carnosine and histidine‐containing dipeptides (HCDs) on mental health outcomes, we reported a significant improvement in depressive symptoms and quality of life with carnosine/HCDs compared with placebo in a mixed population of healthy individuals and those with mental health disorder (Kabthymer et al. 2024). However, there is limited evidence as there are only a few studies, and the results are inconsistent to draw conclusions (Hisatsune et al. 2016; Kabthymer et al. 2024; Katakura et al. 2017; Masuoka et al. 2019; Szczesniak et al. 2014). Importantly, no previous trials have examined the effects of carnosine on depressive symptoms and quality of life in individuals with prediabetes and T2DM.

To address this gap, we conducted a secondary analysis of a randomized controlled trial (RCT) to examine whether carnosine supplementation may improve depressive symptoms and quality of life compared with placebo in individuals with prediabetes or well‐controlled T2DM and without diagnosed clinical depression.

## 2 Materials and Methods

A double‐blind placebo‐controlled parallel group randomized control trial (RCT) was conducted to examine the impact of carnosine supplementation on glycemic control, with the primary outcomes published elsewhere (Hariharan et al. 2024). Detailed study protocol was published elsewhere (Baye et al. 2017). The trial was registered at clinicaltrials.gov (NCT02917928) and was conducted according to the standardized Protocol Interventions: Recommendations for Interventional Trials (SPIRIT) 2013 Statement (Chan et al. 2013) and is reported in line with the CONSORT guidelines (Supporting Information) (Schulz et al. 2010).

### 2.1 Sample Size

The trial sample size was primarily calculated and powered to detect a 20% in fasting glucose (the primary outcome), described in detail elsewhere (Hariharan et al. 2024). Post hoc power calculation was done for the current study using G*power version 3.1.9.7 and determined that a sample size of 38 provides a power of 80% to detect an effect size of 0.47.

### 2.2 Eligibility Criteria

Eligible participants were 18–70 years old with well‐controlled T2DM (HbA1c < 8%) or prediabetes, diagnosed via OGTT per WHO guidelines. Participants with T2DM were either diet‐controlled or taking only metformin. Individuals had a BMI under 40 kg/m^2^ with stable body weight over the past year. Exclusion criteria included mental health disorders, major chronic illnesses, pregnancy, lactation, smoking, or excessive alcohol consumption (> 4 drinks/week for men, > 2 for women). Participants had to maintain their medications, diet, and physical activity routine throughout the 14‐week trial. Those with HbA1c exceeding 8% or requiring medication changes post‐randomization were withdrawn from the study.

### 2.3 Recruitment, Intervention, and Randomization

Participants were recruited through advertisements via posters, radio, email newsletters, and newspapers. Interested individuals completed an eligibility screening by phone, followed by an in‐person screening visit. During this visit, they were assessed against inclusion and exclusion criteria and underwent anthropometric measurements and an OGTT test at the Monash Health Clinical Trials Centre in Melbourne, Australia.

### 2.4 Randomization

Block randomization using block sizes of four by sex was used to randomly allocate eligible participants to intervention or placebo using computer‐generated randomization. The researchers, nurses, outcome assessors, and other personnel were also blinded to the codes. The codes were only revealed by the clinical trials pharmacist after the last participant had completed the trial and primary outcomes were analyzed.

### 2.5 Intervention

The intervention group received 2 g of carnosine daily (1 g twice a day) for 14 weeks, while the placebo group took identical methylcellulose capsules. Both groups were blinded to their allocation. Adverse events were monitored and reviewed by the trial doctor, with follow‐ups as needed. Compliance was assessed by collecting returned supplement containers, and monthly phone check‐ins were conducted to track any side effects, if any.

### 2.6 Outcomes

Outcomes for this study were depressive symptoms and health‐related quality of life as assessed by a self‐administered patient health questionnaire (PHQ‐8) (Kroenke et al. 2009) and five dimension three level EuroQol (EQ‐5D‐3L) (Rabin and de Charro 2001) questionnaire, respectively. The PHQ‐8 questionnaire assesses the frequency of eight symptoms, which include little interest in doing things; feeling down, depressed, or hopeless; trouble falling or staying asleep, or sleeping too much; feeling tired or having little energy; poor appetite or overeating; feeling bad about yourself; trouble concentrating; moving or speaking so slowly or the opposite. Each question is scored from 0 to 3, where 0 indicates “not at all,” 1 for “several days,” 2 for “more than half the days,” and 3 for “nearly every day.” The scores of each question were summed up to calculate the total PHQ‐8 score. Similarly, EQ‐5D‐3L consists of five questions with three‐level responses on morbidity, self‐care, usual activities, pain/discomfort, and anxiety/depression. For pain/discomfort and anxiety/depression the responses were scored as 0 for “none,” 1 for “moderate,” and 2 for “severe.” Whereas for morbidity, self‐care, and usual activities, the responses were scored as 0 for “no problems,” 1 for “some problems,” and 2 for “confined to bed” or “unable to wash or dress myself/perform my usual activities.” The scores of each question were summed up to calculate the total EQ‐5D‐3L score. All outcomes were measured at baseline and repeated after 14 weeks. Health‐related quality of life analyses was conducted at the individual dimension level rather than deriving a composite utility index score. This approach was adopted to explore potential dimension‐specific changes in quality of life outcomes.

### 2.7 Anthropometric, Biochemical, and Body Composition Assessment

The study physician conducted medical history assessments and physical examinations, including anthropometric measurements using a digital scale (Tanita BWB‐600), a standing stadiometer, and vital signs recorded with an Omron digital blood pressure monitor (Model: BBP‐742). Waist and hip circumferences were measured using a tape measure, with waist measurements taken at the midpoint between the upper iliac crest and lowest rib, and hip measurements at the widest part of the buttocks.

### 2.8 Statistical Analysis

Descriptive data were presented as mean ± standard deviation (SD) if data were normally distributed or median with interquartile range (IQR) if the data were skewed. The normality of the data was checked using Shapiro–Wilk tests, and log transformation of the data was done before analysis. The analysis included all participants with available data for both baseline and follow‐up assessments, and there was no imputation for missing data. For within‐group comparisons, paired *t*‐tests were used to compare variables with normal distribution and Wilcoxon's sign rank test if the data were skewed. Changes in outcome variables (follow‐up – baseline) were computed (delta). Analysis of covariance (ANCOVA) models adjusted for baseline values, BMI, and age were used to determine between‐group differences. All tests were two‐tailed, and the significance level was set at *p* ≤ 0.05. Stata version 18 statistical software was used for analysis.

## 3 Result

**FIGURE 1:**
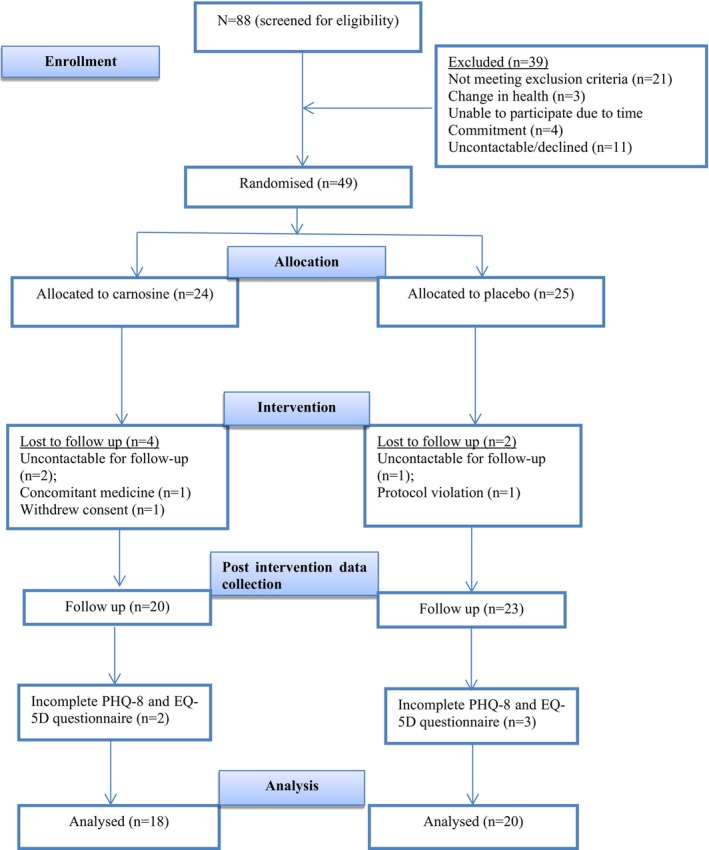
Study flow chart for a randomized controlled trial of the effects of carnosine supplementation on depressive symptoms and health‐related quality of life.

Eighty‐eight participants were assessed for eligibility; 21 did not meet the inclusion criteria, and the remaining 67 attended medical review. Before randomization, 18 participants were excluded based on medical exam; time‐commitment issues, loss of contact, or declined participation. In total, 49 participants were randomly assigned to receive either carnosine (*n* = 24) or placebo (*n* = 25). By the end of the intervention, six participants dropped out or excluded due to protocol violation (*n* = 1), change of medication (*n* = 1), withdrawing consent (*n* = 1) or being uncontactable for follow‐up (*n* = 3). The study was completed by 43 participants; however, five additional participants were excluded due to incomplete data on depression and quality of life scales, leaving 38 individuals (*n* = 18 carnosine; *n* = 20 placebo) for inclusion in this study, analyzed in a per‐protocol fashion. The participant flowchart is shown in Figure 1.

### 3.1 Sample Characteristics

Participants had a median age of 54.8 years (IQR: 46.2, 59.4), with the majority of participants being male (73.6%). The mean ± standard deviation (SD) for weight at baseline was 81.9 ± 23.2 kg, and the mean BMI was 29.0 ± 4.2 kg/m^2^. Nearly half of the participants have a Caucasian ethnic background 18 (47.4%) and are physically active (i.e., moderate exercise more than twice a week; *n* = 22, 57.9%). Nearly half of the participants had type 2 diabetes 18 (47.4%) (Table 1).

**TABLE 1:** Sample demographic and baseline characteristics.

Variables	Total (*n* = 38)	Carnosine (*n* = 18)	Placebo (*n* = 20)
Age, yrs., median (IQR)	54.8 (46.2,59.4)	54.95 (49.5,59.5)	51.8 (42.6,59.3)
Sex, Female *n* (%)	10 (26.3)	4 (22.2)	6 (30)
Weight, kg, mean ± SD	81.9 ± 23.2	87.8 ± 25.2	76.6 ± 20.5
BMI, kg/m^2^, mean ± SD	29.0 ± 4.2	29.6 ± 4.7	28.4 ± 3.7
Ethnicity, *n* (%)			
Caucasian	18 (47.4)	9 (45)	9 (45)
Asian	16 (42.1)	6 (33.3)	10 (50)
Others^a^	4 (10.5)	3 (16.7)	1 (5.0)
Alcohol, *n* (%)			
Never	14 (36.8)	7 (38.9)	7 (35)
Past drinker	3 (7.9)	1 (5.6)	2 (10)
Current drinker	21 (55.3)	10 (55.6)	11 (55)
Physical activity, *n* (%)			
More than twice a week	22 (57.9)	11 (61.1)	11 (55)
Once or twice a week	8 (21.1)	4 (22.2)	4 (20)
Once or twice a month	2 (5.3)	1 (5.6)	1 (5)
Less than once a month	1 (2.6)	0	1 (5)
Never	5 (13.2)	2 (11.1)	3 (15)
Diabetes status, *n* (%)			
T2DM	18 (47.4)	8 (44.4)	10 (50)
Prediabetes	20 (52.6)	10 (55.6)	10 (50)

^a^ Includes African, Middle Eastern, and South American ethnicities.

### 3.2 The Effect of Carnosine Supplementation on Depressive Symptoms

After 14 weeks of intervention, within‐group comparisons showed that carnosine supplementation showed no significant change from baseline (*p* > 0.05) in most parameters, both in the carnosine and placebo groups, except for a significant improvement in appetite (−0.3 ± 0.6, *p* = 0.03) in the carnosine group. Regarding between‐group comparisons, the carnosine group had a statistically significant reduction in depressive symptoms assessed by total PHQ‐8 score compared with placebo (mean difference = −2.0, 95% CI: −3.9, −0.2; *p* = 0.03). However, there were no differences in change values for the eight sub‐components of the PHQ‐8 scale (all *p* > 0.05; Table 2).

**TABLE 2:** The effect of carnosine supplementation on depressive symptoms measured with patient health questionnaire (PHQ‐8).

Variables	Placebo (*n* = 20)				Carnosine (*n* = 18)				Between groups		
	Baseline	Follow up	Δ	P^a^	Baseline	Follow up	Δ	P^a^	Δ^e^ (95% CI)	P^b^	P^c^
Total PHQ_8 score	3.0 ± 2.6	3.7 ± 3.9	0.8 ± 2.8	0.24	3.4 ± 3.2	1.9 ± 1.9	−1.5 ± 2.6	0.03	−2.0 (−3.9, −0.2)	0.08	0.03*
Little interest in doing things	0.2 ± 0.4^d^	0.5 ± 0.9	0.3 ± 0.9	0.29	0.4 ± 0.8	0.2 ± 0.4	−0.2 ± 0.9	0.43	−0.3 (−0.9, 0.2)	0.24	0.25
Feeling down, depressed, or hopeless	0.4 ± 0.6	0.3 ± 0.4	−0.1 ± 0.4	0.31	0.3 ± 0.5	0.1 ± 0.3	−0.2 ± 0.5	0.10	−0.1 (−0.4, 0.1)	0.28	0.28
Trouble falling or staying asleep, or sleeping too much	0.8 ± 0.9	0.6 ± 0.8	−0.2 ± 0.8	0.39	0.5 ± 0.6	0.4 ± 0.8	−0.1 ± 0.9	0.13	0.0 (−0.6, 0.5)	0.54	0.91
Feeling tired or having little energy	0.8 ± 0.8	0.6 ± 0.6	−0.3 ± 0.6	0.09	0.7 ± 0.8	0.6 ± 0.7	−0.1 ± 0.9	0.56	−0.1 (−0.5, 0.3)	0.97	0.68
Poor appetite or overeating	0.3 ± 0.5	0.4 ± 0.6	0.1 ± 0.7	0.94	0.7 ± 0.8	0.3 ± 0.5	−0.3 ± 0.6	0.033	−0.2 (−0.6, 0.2)	0.95	0.26
Feeling bad about yourself	0.2 ± 0.4	0.4 ± 0.6	0.2 ± 0.6	0.17	0.4 ± 0.7	0.2 ± 0.4	−0.2 ± 0.6	0.17	−0.3 (−0.6, 0.1)	0.27	0.12
Trouble concentrating	0.3 ± 0.6	0.2 ± 0.7	−0.1 ± 0.5	0.65	0.2 ± 0.6	0.2 ± 0.4	0.0 ± 0.5	0.65	0.0 (−0.3, 0.4)	0.86	0.88
Moving or speaking so slowly or the opposite	0.1 ± 0.5	0.3 ± 0.7	0.2 ± 0.7	0.54	0.0 ± 0.0	0.1 ± 0.2	0.1 ± 0.2	0.32	−0.1 (−0.5, 0.3)	0.28	0.69

^a^ Determined with the use of paired *t*‐tests.

^b^ Determined with Unadjusted ANCOVA.

^c^ Determined with the use of ANCOVA adjusted for baseline observation, age and BMI.

^d^ Mean ± SD; *p < 0.05; Δ = mean difference (delta).

^e^ Mean change in carnosine group – mean change in placebo group.

### 3.3 The Effect of Carnosine Supplementation on Quality of Life

After 14 weeks of supplementation with carnosine or placebo, there was no significant change from baseline in total EQ‐5D‐3L or the sub‐components of EQ‐5D‐3L in within‐group comparisons for either group (all *p* > 0.05). Similarly, there were no differences in change values between carnosine and placebo groups for both total EQ‐5D‐3L scores and scores of subcomponents of the EQ‐5D‐3L tool (all *p* > 0.05; Table 3).

**TABLE 3:** The effect of carnosine supplementation on health related quality of life (EQ‐5D‐3L).

Variables	Placebo (*n* = 20)				Carnosine (*n* = 18)				Between groups		
	Baseline	Follow up	Δ	P^a^	Baseline	Follow up	Δ	P^a^	Δ^e^ (95% CI)	P^b^	P^c^
Total EQ‐5D score	0.6 ± 1.2^d^	0.6 ± 0.9	0.0 ± 1.3	0.74	0.7 ± 1.2	0.5 ± 1.5	−0.2 ± 2.0	0.24	−0.1 (−1.0, 0.8)	0.90	0.78
Mobility	0.1 ± 0.3	0.1 ± 0.3	0.0 ± 0.4	1.00	0.1 ± 0.2	0.1 ± 0.3	0.1 ± 0.4	0.56	0.0 (−0.25, 0.24)	0.91	0.96
Self care	0.1 ± 0.2	0.1 ± 0.2	0.0 ± 0.2	0.31	0.1 ± 0.2	0.1 ± 0.2	0.0 ± 0.2	1.00	0.1 (−0.3, 0.3)	0.30	0.25
Usual activities	0.1 ± 0.3	0.1 ± 0.2	0.0 ± 0.4	0.56	0.1 ± 0.2	0.1 ± 0.5	0.0 ± 0.5	0.96	0.1 (−0.2, 0.3)	0.60	0.64
Pain discomfort	0.2 ± 0.4	0.3 ± 0.5	0.1 ± 0.4	0.31	0.3 ± 0.5	0.1 ± 0.3	−0.2 ± 0.5	0.18	−0.3 (−0.5, 0.03)	0.16	0.08
Anxiety/depression	0.1 ± 0.3	0.1 ± 0.3	0.0 ± 0.4	1.00	0.3 ± 0.5	0.1 ± 0.3	−0.2 ± 0.6	0.25	0.0 (−0.2, 0.3)	0.91	0.85

^a^ Determined with the use of paired *t*‐tests.

^b^ Determined with Unadjusted ANCOVA.

^c^ Determined with the use of ANCOVA adjusted for baseline observation, age, and BMI.

^d^ Mean ± SD; Δ = mean difference (delta).

^e^ Mean change in carnosine group – mean change in placebo group.

## 4 Discussion

We conducted the first randomized controlled trial to examine the effects of carnosine supplementation on depressive symptoms and health‐related quality of life among patients with prediabetes and well‐controlled type 2 diabetes without a diagnosis of depression. There was a significant reduction in depressive symptoms based on PHQ‐8 scores in the carnosine group, compared with placebo. We found no significant change between or within groups in health‐related quality of life (EQ‐5D‐3L) total scores or in sub‐components of either the PHQ‐8 or the EQ‐5D‐3L.

The findings of our study are in agreement with previous studies including our previous meta‐analysis (Kabthymer et al. 2024), where carnosine and HCD supplementation were shown to reduce depressive symptoms measured with Becks depression scale. Conversely, no significant change in depressive symptoms was reported in a study among older adults in Japan (Katakura et al. 2017; Masuoka et al. 2019), following 750 mg/day of anserine and 250 mg/d of carnosine supplementation for 12 weeks. In addition, a study by Masuoka et al. (Masuoka et al. 2019) included elderly participants with mild cognitive impairment and found no significant change in geriatric depression scale (GDS) score. Discrepancies between our findings and these prior studies may be due to differences in the age and health status of participants, as the latter were restricted to older adults (> 65 years) and were non‐diabetic, whereas participants in the current study were younger (median age = 54.8 years) with prediabetes or T2DM. Indeed, depression is more prevalent in later life due to medical comorbidities (Szymkowicz et al. 2023) and may not be sufficiently improved by carnosine/HCDs alone.

The observed reduction in depressive symptoms following carnosine supplementation may be attributable to its well‐reported anti‐inflammatory and antioxidant properties (Aldini et al. 2021; Kim et al. 2011). Carnosine acts as an anti‐inflammatory and antioxidant agent by activating the nuclear factor erythroid 2‐related factor 2 (NRF2) signal (Aldini et al. 2021). NRF2 signaling modulates inflammatory mediators, impacting NF‐κB and MAPK pathways, which play a crucial role in controlling inflammation (Saha et al. 2020) and may help reduce neuro‐inflammation‐related depressive symptoms (Kazmi et al. 2024; Mohamed et al. 2024; Wang et al. 2024). Carnosine functions as an antioxidant, neutralizing oxidative stressors such as H₂O₂ and HOCl, while also preventing AGE and ALE formation, offering protection in oxidative‐related conditions like diabetes (Aldini et al. 2021; Kasamatsu et al. 2021; Menini et al. 2020). It is well‐documented that individuals with depression tend to have elevated levels of systemic inflammation and oxidative (Kohler et al. 2017; Osimo et al. 2019). Findings from a previous meta‐analysis by our group highlighted that carnosine/HCDs supplementation might lead to a reduction in serum inflammatory and oxidative stress biomarkers such as C‐reactive protein (CRP), tumor necrosis factor‐α (TNF‐α) and malondialdehyde (MDA) (Saadati et al. 2023).

Thus, the anti‐inflammatory and anti‐oxidative properties of carnosine may be responsible for reducing depressive symptoms. While plausible, however, the exact mechanism by which carnosine may reduce depressive symptoms, and whether this occurs via inflammatory or oxidative pathways or both, remains unknown.

On the other hand, improvements in depressive symptoms may be related to improved blood glucose levels in response to carnosine supplementation (Badescu et al. 2016). We and others have reported that carnosine lowers blood glucose in prediabetes and T2DM (de Courten et al. 2016; Hariharan et al. 2024; Matthews et al. 2021; Menon et al. 2020), supporting this potential mechanism of action. Since depressive symptoms are more common in individuals with T2DM (Yang et al. 2022), carnosine might be a beneficial adjunctive therapy for concurrent improvement in glycemic control and depressive symptoms in those with prediabetes and T2DM; however, the efficacy of carnosine in this context requires further study.

Finally, we found no significant change in health‐related quality of life measured with the EQ‐5D‐3L questionnaire after carnosine supplementation. Our finding contrasts a previous meta‐analysis that reported improved quality of life measured with the short‐form survey (SF‐36) questionnaire (Kabthymer et al. 2024). A possible explanation for this discrepancy may relate to differences in the tools used and duration of intervention. The recall period in particular varies, whereby the EQ‐5D‐3L assesses the health status of the day of instrument administration, while SF‐36 assesses the health status for the past 30 days (Kularatna et al. 2019). Furthermore, when compared with SF‐36, EQ‐5D‐3L tends to have a higher ceiling effect, which results in a reduced ability to detect changes in relatively young, healthy individuals with a good baseline quality of life, such as the cohort in the present study (Kularatna et al. 2019; Xie et al. 2022).

This study has several strengths. Notably, this is the first study to assess the role of carnosine on depressive symptoms in patients with prediabetes and T2DM, thus contributing novel evidence to this area of research. We used a randomized, double‐blind, placebo‐controlled design representing the gold standard for establishing causality. We also used a higher dose of carnosine compared to the majority of previous trials, thereby increasing the potential efficacy of the intervention. However, some limitations are acknowledged, including the secondary nature of our analysis, where the trial was not powered to detect changes in depressive symptoms or quality of life outcomes. Our sample size may, therefore, be too small, and additional investigations in larger studies are warranted, particularly to verify the non‐significant results for the EQ‐5D‐3L quality of life metric; use of EQ‐5D‐3L has only 3 levels resulting in relatively low sensitivity that might explain the insignificant association reported in quality of life (Afshari et al. 2022). The intervention had a duration of 14 weeks – a duration that, while reasonable, may be insufficient to capture meaningful changes in depressive symptoms or quality of life. Our study cohort also had no prior history of depression and low PHQ‐8 scores at baseline, suggesting that any treatment effect would likely be small, thus requiring a larger sample size to detect significant differences. Notwithstanding these limitations, this study demonstrates significant differences in total PHQ‐8 scores between carnosine and placebo groups. This finding underscores the potential role of carnosine in alleviating depressive symptoms in individuals with prediabetes or T2DM, supporting the need for further, well‐powered clinical trials to corroborate these results.

## 5 Conclusion

Carnosine supplementation resulted in reduced depressive symptoms but no improvement in health related quality of life in individuals with prediabetes and well‐controlled T2DM without a history of depression. These findings highlight then the need for further studies with larger sample sizes in diverse populations to clarify the role of carnosine in mental health and quality of life outcomes among these high‐risk populations.

## Author Contributions

**Robel Hussen Kabthymer:** data curation, formal analysis, investigation, methodology, software, visualization, writing – original draft, writing – review and editing; **Jack Feehan:** formal analysis, investigation, methodology, software, visualization, validation, writing – review and editing; **Aya Mousa:** investigation, methodology, software, visualization, validation, writing – review and editing; **Giancarlo Aldini:** conceptualization, validation, writing – review and editing; **Maximilian de Courten:** conceptualization, validation, writing – review and editing, funding acquisition; **James Cameron:** conceptualization, resources, validation, writing – review and editing, funding acquisition; **Barbora de Courten:** conceptualization, methodology, investigation, resources, supervision, validation, writing – review and editing, funding acquisition. All authors have read and approved the final manuscript.

## Funding

We would like to thank the Royal Australasian College of Physicians (RACP) and the CASS foundation for the financial support. RHK is supported by a Monash International Tuition Scholarship (MITS) and a Monash Graduate Scholarship (MGS). AM is supported by a fellowship from the National Health and Medical Research Council (NHMRC) of Australia.

## Ethics Statement

This study was conducted in accordance with the Helsinki Declaration and registered at clinicaltrials.gov (NCT02917928). The study was ethically approved by the Human Research Ethics Committee of Monash Health (Ref. No. 16061A) and Monash University (ID: 7787). All participants signed written informed consent before joining the trial.

## Conflicts of Interest

The authors declare no conflicts of interest.

## Supporting information


**Supporting Information S1:** CONSORT guideline checklist.

## Data Availability

The data that support the findings of this study are available from the corresponding author upon reasonable request.
